# Efficacy of Olanzapine Combined Therapy for Patients Receiving Highly Emetogenic Chemotherapy Resistant to Standard Antiemetic Therapy

**DOI:** 10.1155/2015/956785

**Published:** 2015-09-03

**Authors:** Masakazu Abe, Yuka Kasamatsu, Nobuhiro Kado, Shiho Kuji, Aki Tanaka, Nobutaka Takahashi, Munetaka Takekuma, Yasuyuki Hirashima

**Affiliations:** Division of Gynecology, Shizuoka Cancer Center Hospital, 1007 Shimonagakubo, Nagaizumi-cho, Sunto-gun, Shizuoka 411-8777, Japan

## Abstract

*Objective*. Olanzapine is proved to be effective for chemotherapy induced nausea and vomiting (CINV). But its efficacy in combination with standard antiemetic therapy is unknown. The purpose of this study is to prove the preventive effect of olanzapine for the prevention of CINV caused by highly emetogenic chemotherapy when used with standard antiemetic therapy. *Method*. Gynecologic cancer patients receiving cisplatin-based chemotherapy who had grade 2 or 3 nausea in overall phase (0–120 h after chemotherapy) despite standard therapy were assigned to this study. From the next cycles to cycles in which patients developed grade 2 or 3 nausea, they received olanzapine with standard therapy. 5 mg oral olanzapine was administered for 7 days from the day before chemotherapy. The effectiveness of preventive administration of olanzapine was evaluated retrospectively. The primary endpoint was nausea control rate (grade 0 or 1) with olanzapine. *Results*. Fifty patients were evaluable. The nausea control rate with olanzapine was improved from 58% to 98% in acute phase (0–24 h after chemotherapy) and 2% to 94% in delayed phase (24–120 h after chemotherapy). In overall phase, the nausea control rate improved from 0% to 92%, and it was statistically significant (*P* < 0.001). *Conclusion*. Preventive use of olanzapine combined with standard antiemetic therapy showed improvement in control of refractory nausea.

## 1. Introduction

Chemotherapy induced nausea and vomiting (CINV) is one of the most harmful adverse effects even though there is a significant progress in antiemetics nowadays. CINV could bring anorexia, malnutrition, dehydration, and anxiety toward chemotherapy to patients and for that it is important to reduce symptoms of CINV as possible. National Comprehensive Cancer Network (NCCN), American Society of Clinical Oncology (ASCO), and Multinational Association of Supportive Care in Cancer (MASCC) have developed antiemetic guidelines based on evidence. In Japan, the first guideline for proper use of antiemetics was provided by Japan Society of Clinical Oncology in 2010 based on guidelines written above.

These guidelines recommend triple therapy consisted of 5-HT3 receptor antagonist, NK-1 receptor antagonist, and dexamethasone as a standard antiemetic therapy toward highly emetogenic chemotherapy (HEC) [1, 2]. Multiple reports proved the effect of this therapy [3–6]. Complete response (no vomiting, no rescue, and any nausea) to HEC is reported to be around 80% in acute phase (0–24 h after chemotherapy) and 60–70% in delayed phase (24–120 h after chemotherapy). However, there is no effective therapy toward CINV which is resistant to standard antiemetics reported.

In guidelines above, olanzapine, the atypical antipsychotic, is mentioned as a usable agent for CINV refractory for standard antiemetic therapy. Olanzapine is reported to be equal or more effective for CINV compared to aprepitant and dexamethasone [7, 8]. Moreover, olanzapine is reported as an effective and tolerable agent which can be used as a salvage therapy to standard therapy refractory CINV [9]. However, preventive administration of olanzapine for standard therapy refractory CINV has not been proved effective or safe so far. In this study, we administered olanzapine with standard antiemetic therapy as a preventive therapy to patients treated with HEC containing cisplatin who had grade 2 or 3 nausea (Common Terminology Criteria for Adverse Events; CTCAE ver. 4.0) in spite of receiving standard antiemetic therapy. The control of nausea and vomiting was evaluated retrospectively.

## 2. Methods

### 2.1. Patients

Fifty patients were assigned to this study. They were gynecological cancer patients who were treated with HEC regimen containing cisplatin and had symptoms of grade 2 or 3 nausea in overall phase (0–120 h after chemotherapy) in spite of receiving standard antiemetic therapy. There were 32 patients of grade 3 and 18 patients of grade 2. All patients were informed of drug information and the consent of using olanzapine was obtained. Since olanzapine is contraindicated in patients with diabetes mellitus, their blood sugar level and hemoglobin A1c were checked to confirm that they do not have glucose intolerance.

Regimens with less than 50 mg/m^2^ cisplatin were included in this study because ASCO, MASCC, and Japanese guidelines include these regimens in HEC although NCCN classifies them as moderate emetogenic chemotherapy (MEC).

We conducted this study in accordance with ethical principles based on the Declaration of Helsinki. All the data and information of patients were processed considering privacy and patients were not identifiable.

### 2.2. Treatment Plans

As a standard antiemetic therapy, 5-HT3 receptor antagonist (palonosetron 0.75 mg or granisetron 3 mg on day 1), NK-1 receptor antagonist (aprepitant 125 mg on day 1, 80 mg on days 2-3), and dexamethasone (9.9 mg on day 1, 6.6 mg on days 2–4) were administered. Olanzapine was given with standard antiemetic therapy from the cycles next to cycles in which patients developed grade 2 or 3 nausea in overall phase though they were treated with standard antiemetic therapy. 5 mg of oral olanzapine was given for 7 days starting at the day before cisplatin was administered.

### 2.3. Parameters Assessed

The grades of nausea through acute phase, delayed phase, and overall phase were evaluated according to medical record written by doctors, nurses, and pharmacists using CTCAE ver. 4.0. The primary endpoint was nausea control rate. It was defined as the rate of patients whose grade of nausea was controlled within 0 or 1. The secondary endpoints were no vomiting rate (the rate of patients who did not have any vomiting), complete response rate (no vomiting, no rescue, and any nausea), complete control rate (no vomiting, no rescue, and nausea grade 0 or 1), and total control rate (no vomiting, no rescue, and no nausea). We compared cycles containing only standard antiemetic therapy with cycles containing both standard antiemetic therapy and olanzapine. Adverse effects and laboratory data were evaluated based on CTCAE ver. 4.0.

### 2.4. Statistical Analysis

We compared cycles in which patients developed grade 2 or 3 nausea with standard therapy and cycles in which they received olanzapine with standard therapy for the first time. We used McNemar test to evaluate the improvements in each parameter. *P* < 0.05 was considered statistically significant in this study.

## 3. Results

Patient's characteristics are shown in Table 1. Regimens with cisplatin more than 50 mg/m^2^ were used in 45 patients and regimens with cisplatin less than 50 mg/m^2^ were used in 5 patients. In FP therapy, weekly CDDP, and weekly TP therapy, radiation therapy (external pelvic irradiation, 1.8 Gy/day) was used simultaneously. The mode of the number of cycles in which patients developed grade 2 or 3 nausea though they were treated with standard antiemetic therapy was cycle 1 and there were 29 cases.

The changes of nausea grades with the usage of olanzapine are shown in Table 2. There were no patients who had heavier nausea. In most of the patients, their nausea improved after they started to use olanzapine.

The nausea control rate is shown in Table 3. The nausea control rate with olanzapine improved from 58% to 98% in acute phase and 2% to 94% in delayed phase. In overall phase, the nausea control rate improved from 0% to 92%, and it was statistically significant (*P* < 0.001).

No vomiting rate, no rescue therapy rate, complete response rate, complete control rate, and total control rate of cycles before using olanzapine and those with olanzapine are shown in Table 4. In the cycle where patients developed grade 2 or 3 nausea, 19 patients vomited and 49 had rescue therapy in overall phase. As this result, complete response rate of overall phase in group of patients without olanzapine was only 2%. No vomiting rates of cycles using olanzapine in acute phase, delayed phase, and overall phase were 100%, 96%, and 96%, respectively. In each phase, improvement was statistically significant (*P* < 0.05). No rescue therapy rates of cycles using olanzapine in acute phase, delayed phase, and overall phase were 98%, 82%, and 82%, respectively. In each phase, improvement was statistically significant (*P* < 0.001). Complete response rate and complete control rate of cycles using olanzapine were 82–98% in all phases and they improved significantly compared with cycles without olanzapine (*P* < 0.001). Total control rate using olanzapine was 86% in acute phase, 42% in delayed phase, and 40% in overall phase, but all rates improved significantly (*P* < 0.001).

As adverse effects, grade 1 or 2 drowsiness was seen in 26 patients. There were 18 patients of grade 1 (36%) and 8 patients of grade 2 (16%). Six patients had to reduce the dose of olanzapine to 2.5 mg because of grade 2 drowsiness, but no patients had to stop taking it. There were no grade 3-4 adverse effects. Forty-nine patients out of 50 wished to continue taking olanzapine and used olanzapine through whole cycles of chemotherapy. One patient had to stop receiving chemotherapy because of the progression.

## 4. Discussion

As far as we know, this is the first study to report the preventive effect of olanzapine used with standard antiemetic therapy toward CINV caused by highly emetogenic chemotherapy.

First, the most important point in this study is that using olanzapine combined with standard antiemetic therapy was effective for preventing nausea and vomiting in patients with CINV resistant to standard antiemetic therapy. Although olanzapine was given to patients who had grade 2 or 3 nausea despite standard antiemetic therapy, the nausea control rate was improved more than 90% in those patients by combined use of olanzapine. The improvement was statistically significant. This shows the possibility of preventive administration of olanzapine becoming a new effective choice toward CINV resistant to standard antiemetic therapy.

Second, the nausea and vomiting were also controlled in delayed phase in almost the same level of acute phase, although delayed phase is known to be more difficult in terms of controlling these symptoms. It is very interesting that the nausea control rate was 94% in delayed phase while it was 98% in acute phase. There is a possibility that olanzapine is effective to the mechanism of nausea in delayed phase which is refractory to standard antiemetic therapy. Even in patients who had grade 2 or 3 nausea, complete response rate was 82% in delayed phase because both the nausea control rate and the vomiting control rate significantly improved.

It is reported that complete response rate of standard antiemetic therapy is 80% in acute phase and 60–70% in delayed phase [3–6], which are thought to be relatively good results. However we must pay attention to the fact that these studies include both male and female patients. The results with a group consisted of only female patients are worse compared to those with a group consisted of both sexes because women have higher risk of CINV. In particular, complete response rate in delayed phase is as low as 50% in gynecologic cancer patients [10]. In phase III randomized control trial which compared the effect of antiemetic therapy toward cisplatin-based chemotherapy between both genders, there were no differences in male patients and female patients in first cycle when they were treated with triple therapy. The percentage of patients who had no emesis was 70% in both genders. However, the difference of effect in gender became bigger as they continued the chemotherapy. The percentage of the female patients with no emesis in 6th course of chemotherapy was only 44% where 60% of male patients had no emesis [11]. Therefore we need stronger antiemetic therapy in female patients and that is the reason we have expectation in efficacy of olanzapine.

Olanzapine is classified as an atypical antipsychotic and it is used to treat schizophrenia and bipolar disorders. Olanzapine is called MARTA (multi-acting-receptor-targeted antipsychotics) and its main characteristic is that it is an antagonist of multiple chemoreceptors such as dopamine (D1, D2, D3, D4, and D5), serotonin (5-HT2a, 5-HT2c, 5-HT3, and 5-HT6), histamine (H1), adrenalin (*α*1), and acetylcholine-muscarine (Achm1–Achm5) [12]. Olanzapine is not originally antiemetic agent, but, due to its strong antiemetic effect, there are many studies reporting its efficacy toward CINV, nausea due to opioids, and nausea and vomiting in terminal stage in patients with malignant tumors.

Acetylcholine-muscarine (Achm), dopamine (D2), histamine (H1), serotonin (5-HT2, 5-HT3), and neurokinin-1 (NK-1) are known as main neurotransmitters related to CINV. There are chemoreceptors of these transmitters in central nervous system. There are H1 and Achm in vestibular apparatus, 5-HT3, NK-1, and D2 in CTZ, and 5-HT2, 5-HT3, NK-1, D2, H1, and Achm in vomiting center and it is thought that the network between these receptors causes nausea and vomiting [13]. Olanzapine is a medication which can be an antagonist of those 4 receptors except NK-1 receptor and related to all of the vestibular apparatus, CTZ, and vomiting center. Theoretically, by using both standard antiemetic therapy and olanzapine, all chemoreceptors affecting CINV can be blocked because olanzapine can be an antagonist of chemoreceptors which cannot be blocked using only standard antiemetic therapy. Also, olanzapine is known to have less adverse effects such as extra pyramidal symptoms and akathisia compared with conventional antipsychotics (prochlorperazine, haloperidol, etc.) and metoclopramide which have been used for CINV [14].

There are several phase III randomized control trials on efficacy of olanzapine towards CINV. In the study which compared the olanzapine with aprepitant in patients using regimen containing cisplatin or AC therapy (doxorubicin, cyclophosphamide), complete control rates were almost the same in both acute phase and delayed phase. But rate of patients who had no nausea at all was 69% in olanzapine group while it was 38% in aprepitant group [7]. Therefore olanzapine was proved to be comparable or even more effective compared with aprepitant. The study compared olanzapine and dexamethasone with patients using HEC or MEC; complete control rates in acute phase were almost the same in both groups. However in delayed phase, olanzapine group had significantly better complete response rate in both HEC and MEC regimens (HEC: nausea 69% versus 30%, vomiting 78% versus 56%; MEC: nausea 83% versus 58%, vomiting 89% versus 75%) [8]. In the study which compared olanzapine with metoclopramide used as salvage therapy for patients who had CINV resistant to standard antiemetic therapy, the rates of patients without vomiting were 70% versus 31% and the rates of those without nausea were 68% versus 23% within 72 hours after salvage. By this result, olanzapine was proved to be a stronger salvage therapy agent [9]. There were no grade 3 or 4 adverse effects in these studies.

The limitation of this study is that, firstly, this is a retrospective before-after comparative study with only small number of patients. A prospective study should be conducted in the future. Second, the evaluation of nausea was done by doctors, nurses, and pharmacists based on objective indicator. We believe that there is no big divergence with the self-evaluations of patients but to evaluate the true therapeutic effect, evaluation tool such as patient diary is needed as subjective self-evaluation. Third, we do not know the optimal dose of olanzapine yet. The rate of drowsiness was quite high such as 52% in this study, so there were 6 patients who had their olanzapine reduced to 2.5 mg. Meanwhile, there was one patient who had to take 10 mg of olanzapine due to strong nausea. We have to verify the optimal dose of olanzapine used with standard antiemetic therapy. Finally, there are only gynecological cancer patients in this study. We also have to verify if this is also effective for patients using regimens for other kinds of malignant tumors.

We now have an ongoing prospective phase II trial to prove the efficacy and safety of olanzapine used with standard antiemetic therapy toward CINV caused by HEC.

## 5. Conclusion

We treated patients using cisplatin containing HEC regimen with 5 mg of olanzapine who had grade 2 or 3 nausea although they were receiving standard antiemetic therapy. Using olanzapine combined with standard antiemetic therapy improved nausea control rate to more than 90% and it was statistically significant. There was grade 1 or 2 drowsiness in half of the patients but it was feasible.

It is suggested that using olanzapine as a preventive antiemetic agent combined with recommended standard antiemetic therapy could be useful antiemetic regimen. Moreover, this could improve quality of life in patients with cancer who are receiving chemotherapy.

## Figures and Tables

**Table 1:** Characteristics of patients.

Variables		
Age^*^	53 ± 12.3 years	(26–71 years)
Gender		
Male	0	
Female	50	
ECOG performance status		
0	24	
1	18	
2	8	
Height^**^	155.6 ± 3.7 cm	(150–162 cm)
Body weight^**^	55.1 ± 11.2 kg	(42–84 kg)
Body mass index^**^	22.8 ± 4.4	(16.2–33.8)
The cycle in which patients developed grade 2 or 3 nausea despite standard antiemetic therapy		
1	29	
2	11	
3	6	
4	4	
Type of cancer		
Uterine cervical cancer	23	
Uterine corpus cancer	22	
Uterine carcinosarcoma	2	
Ovarian cancer	2	
Vaginal cancer	1	
Regimen of anticancer chemotherapy		
AP (adriamycin 60 mg/m^2^, cisplatin 50 mg/m^2^)	22	
CPT-11/CDDP (irinotecan 60 mg/m^2^, cisplatin 60 mg/m^2^)	8	
CDDP (cisplatin 50 mg/m^2^)	5	
FP (5-fluorouracil 700 mg/m^2^ × 4 days, cisplatin 70 mg/m^2^)^***^	3	
TP (paclitaxel 135 mg/m^2^, cisplatin 50 mg/m^2^)	3	
EP (etoposide 100 mg/m^2^ × 3 days, cisplatin 80 mg/m^2^)	2	
IP (ifosphamide 1.5 g/m^2^ × 4 days, cisplatin 80 mg/m^2^)	2	
weekly CDDP (cisplatin 40 mg/m^2^)^***^	4	
weekly TP (paclitaxel 50 mg/m^2^, cisplatin 30 mg/m^2^)^***^	1	

ECOG: Eastern Cooperative Oncology Group; CDDP: cisplatin.

^*^Median ± S.D. (range).

^**^Mean ± S.D. (range).

^***^Combined with external pelvic irradiation (1.8 Gy/day).

**Table 2:** The changes in nausea grade before/after using olanzapine.

	Acute phase (0–24 h)				Delayed phase (24–120 h)				Overall phase (0–120 h)			
	Nausea grade with OLN				Nausea grade with OLN				Nausea grade with OLN			
	0	1	2	3	0	1	2	3	0	1	2	3
Nausea grade without OLN												
0	17	0	0	0	0	0	0	0	0	0	0	0
1	9	3	0	0	1	0	0	0	0	0	0	0
2	13	1	0	0	6	13	1	0	6	11	1	0
3	4	2	1	0	14	13	2	0	14	15	3	0

OLN: olanzapine.

**Table 3:** Changes of nausea grade and nausea control rate after receiving olanzapine combined with standard antiemetic therapy.

	Acute phase (0–24 h)		Delayed phase (24–120 h)		Overall phase (0–120 h)	
	Without OLN	With OLN	Without OLN	With OLN	Without OLN	With OLN
Nausea grade						
0	17 (34%)	43 (86%)	0 (0%)	21 (42%)	0 (0%)	20 (40%)
1	12 (24%)	6 (12%)	1 (2%)	26 (52%)	0 (0%)	26 (52%)
2	14 (28%)	1 (2%)	20 (40%)	3 (6%)	18 (36%)	4 (8%)
3	7 (14%)	0 (0%)	29 (58%)	0 (0%)	32 (64%)	0 (0%)

Nausea control rate^*^	58%	98%	2%	94%	0%	92%

*P* value^**^	*P* < 0.001		*P* < 0.001		*P* < 0.001	

OLN: olanzapine, ^*^nausea grade 0 or 1 (CTCAE ver. 4.0), and ^**^McNemar test.

**Table 4:** Changes of complete response rate, complete control rate, and total control rate after receiving olanzapine combined with standard antiemetic therapy.

	Acute phase (0–24 h)			Delayed phase (24–120 h)			Overall phase (0–120 h)		
	Without OLN	With OLN	*P* value^*^	Without OLN	With OLN	*P* value^*^	Without OLN	With OLN	*P* value^*^
No vomiting	44 (88%)	50 (100%)	0.03	35 (70%)	48 (96%)	<0.001	31 (62%)	48 (96%)	<0.001
No rescue therapy	27 (54%)	49 (98%)	<0.001	1 (2%)	41 (82%)	<0.001	1 (2%)	41 (82%)	<0.001
CR	26 (52%)	49 (98%)	<0.001	1 (2%)	41 (82%)	<0.001	1 (2%)	41 (82%)	<0.001
CC	24 (48%)	49 (98%)	<0.001	0 (0%)	41 (82%)	<0.001	0 (0%)	41 (82%)	<0.001
TC	17 (34%)	43 (86%)	<0.001	0 (0%)	21 (42%)	<0.001	0 (0%)	20 (40%)	<0.001

^*^McNemar test.

OLN: olanzapine.

CR: complete response (no vomiting, no rescue, and any nausea).

CC: complete control (no vomiting, no rescue, and nausea grade 0 or 1).

TC: total control (no vomiting, no rescue, and nausea grade 0).
